# A survey among German-speaking radiation oncologists on PET-based radiotherapy of prostate cancer

**DOI:** 10.1186/s13014-021-01811-8

**Published:** 2021-05-01

**Authors:** Marco M. E. Vogel, Sabrina Dewes, Eva K. Sage, Michal Devecka, Jürgen E. Gschwend, Matthias Eiber, Stephanie E. Combs, Kilian Schiller

**Affiliations:** 1 Department of Radiation Oncology, Klinikum rechts der isar, Technical University of Munich (TUM), Ismaninger Strasse 22, 81675 Munich, Germany; 2 Department of Radiation Sciences (DRS), Institute for Radiation Medicine (IRM), Helmholtz Zentrum München, Neuherberg, Germany; 3 Department of Urology, Klinikum rechts der Isar, Technical University of Munich (TUM), Munich, Germany; 4 Department of Nuclear Medicine, Klinikum rechts der Isar, Technical University of Munich (TUM), Munich, Germany; 5 Deutsches Konsortium für Translationale Krebsforschung (DKTK), Partner Site Munich, Munich, Germany

**Keywords:** PSMA-PET, Prostate cancer, Radiotherapy, Survey

## Abstract

**Background:**

Positron emission tomography-(PET) has evolved as a powerful tool to guide treatment for prostate cancer (PC). The aim of this survey was to evaluate the acceptance and use of PET—especially with prostate-specific membrane antigen (PSMA) targeting tracers—in clinical routine for radiotherapy (RT) and the impact on target volume definition and dose prescription.

**Methods:**

We developed an online survey, which we distributed via e-mail to members of the German Society of Radiation Oncology (DEGRO). The survey included questions on patterns of care of RT for PC with/without PET. For evaluation of doses we used the equivalent dose at fractionation of 2 Gy with α/β = 1.5 Gy [EQD2(1.5 Gy)].

**Results:**

From 109 participants, 78.9% have the possibility to use PET for RT planning. Most centers use PSMA-targeting tracers (98.8%). In 39.5%, PSMA-PET for biochemical relapse after prior surgery is initiated at PSA ≥ 0.5 ng/mL, while 30.2% will perform PET at ≥ 0.2 ng/mL (≥ 1.0 ng/mL: 16.3%, ≥ 2.0 ng/mL: 2.3%, regardless of PSA: 11.7%). In case of PET-positive local recurrence (LR) and pelvic lymph nodes (LNs), 97.7% and 96.5% of the participants will apply an escalated dose. The median total dose in EQD2(1.5 Gy) was 70.00 Gy (range: 56.89–85.71) for LR and 62.00 Gy (range: 52.61–80.00) for LNs. A total number of ≤ 3 (22.0%) or ≤ 5 (20.2%) distant lesions was most often described as applicable for the definition as oligometastatic PC.

**Conclusion:**

PSMA-PET is widely used among German radiation oncologists. However, specific implications on treatment planning differ among physicians. Therefore, further trials and guidelines for PET-based RT are warranted.

**Supplementary Information:**

The online version contains supplementary material available at 10.1186/s13014-021-01811-8.

## Background

Positron emission tomography (PET) imaging is an important imaging modality for treatment planning in cancer patients especially in those with prostate cancer (PC). Several radiolabeled tracers have been developed and used for patients with PC. Most recently different ligands of the prostate-specific membrane antigen (PSMA) have been introduced and are widely used in clinical practice especially in Germany.

PSMA-targeting PET-tracers allow for identifying tumor lesions with detection rates of 58% at prostate-specific antigen (PSA) levels as low as 0.2–1.0 ng/mL for [68Ga]PSMA, increasing with higher PSA values [1]. This led to a shift in the treatment options of radiotherapy (RT) for PC [2]. Whereas in the past, RT to the prostate, prostate bed or other PC lesions was performed mostly without a morphologic imaging correlate, today precise RT of the true tumor mass with PET imaging is possible. Therefore, the concept of metastasis-directed therapy (MDT) has been introduced [3].

The recently published proPSMA trial showed a 27% higher accuracy for PSMA-PET/computed tomography (CT) compared to conventional imaging with CT and bone scan for staging prior to definitive treatment [4]. Further, PSMA-PET for patients with biochemical recurrence after prior definitive treatment is recommended in the European [5] and German [6] guidelines.

However, PET-based RT planning and treatment differs significantly among centers. Zschaeck et al. reported differing patterns of care for PSMA-PET-based RT in seven university centers [7].

We have designed a survey on patterns of care of RT with/without PET imaging for patents with PC. In the present manuscript we present the results of the second part of the survey on the application of PET imaging which adds novel and unprecedented information on the day-to-day routine of PET-based RT. We sought to determine the use and experience with PET for RT planning, predominantly PSMA-PET, among German-speaking radiation oncologists and the impact PET imaging has on target volume definition and dose prescription.

## Methods

The authors developed a questionnaire with 35 items on RT planning with/without PET imaging for definitive and postoperative treatment of PC as well as oligorecurrent/oligometastatic PC. Questions were created either as single-choice questions, multiple-choice questions, or free-response questions. We included four general epidemiologic questions, six general questions on definitive RT, six general questions on adjuvant/salvage RT, 14 questions on PET-based RT planning, and five questions on RT for oligorecurrent/oligometastatic PC. A team of radiation oncologists and specialists in nuclear medicine reviewed the survey and applied minor changes to enhance usability and readability. For the distribution of the questionnaire, we used the online platform survio.com. We sent a hyperlink via e-mail to all registered members of the German Society of Radiation Oncology (DEGRO). The participation was voluntary and anonymous. The survey was available for completion between March 3rd, 2020 and April 3rd, 2020. The second part of the survey is analyzed within the present manuscript focusing on PET-based RT of PC (see Additional file 1). All aspects focusing on the daily practice patterns of PC treatment are not part of this manuscript.

When participants answered questions for total doses and single doses with dose ranges, we chose the lower end of range for analysis. Brachytherapy doses were not considered in the calculation of the median doses and were stated separately. We excluded the dose values if total dose and single dose did not match since this was most likely due to an input error. For evaluation of the doses we used the equivalent dose at fractionation of 2 Gy (EQD2), calculated using the linear quadratic model with α/β prostate = 1.5 Gy [EQD2(1.5 Gy)]. All statistical analyses were performed using SPSS version 25 (IBM, Armonk, New York, USA).

## Results

Between March 3rd, 2020 and April 3rd, 2020, 109 participants completed the survey. The characteristics of participants are shown in Table 1.

**Table 1:** Characteristics of participants n = 109

	n (%)
*Participants’ institution*	
University hospital	29 (26.6%)
Non-university hospital	26 (23.9%)
Outpatient care center (MVZ)	37 (33.9%)
Medical practice	17 (15.6%)
*Participants’ position*	
Resident	10 (9.2%)
Fellow/specialist	45 (41.3%)
Consultant/chair	54 (49.5%)
*Available RT techniques*	
3D-CRT	100 (91.7%)
IMRT/VMAT	108 (99.1%)
Helical IMRT	26 (23.9%)
IGRT	97 (89.0%)
Stereotactic RT	80 (73.4%)
Proton/heavy ion RT	2 (1.8%)
Brachytherapy	64 (58.7%)

*n* number, *3D-CRT* three-dimensional conventional radiotherapy, *IMRT* intensity-modulated radiotherapy, *VMAT* volumetric arc therapy, *IGRT* image-guided radiotherapy, *RT* radiotherapy

**Fig. 1:**
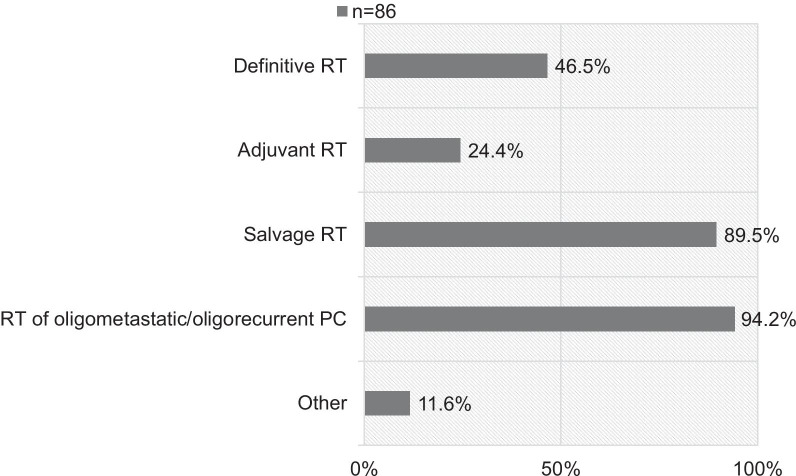
Clinical indications of use for PET imaging in patients with PC (n = 86, multiple choice possible, *PET* positron emission tomography, *RT* radiotherapy, *PC* prostate cancer)

A total of 109 members responded with 78.9% (86/109) having access to PET for RT planning, either in-house (44.0%, 48/109) or with an external cooperation partner (34.9%, 38/109). PSMA-targeting (98.8%, 85/86) and choline agents (15.1%, 15/86) are used. PET/CT (100%, 86/86) and PET/ magnetic resonance imaging (MRI) (34.9%, 30/86) are performed. Most participants (69.8%, 60/86) report problems with the cost coverage of PSMA-PET imaging by health insurance. 96.5% (83/86) of the radiation oncologists who have the possibility to use PET imaging for RT planning do so in the daily clinical routine. Figure 1 shows the clinical indications of use for PET imaging.

**Fig. 2:**
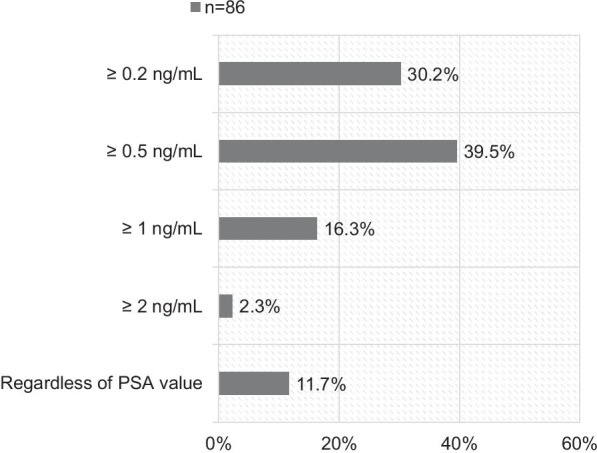
Thresholds of PSA before PET imaging in case of biochemical relapse after prior surgery (n = 86, *PSA* prostate-specific antigen, *ng/mL* nanogram/milliliter)

In case of biochemical relapse after prior surgery, most participants (39.5%) will initiate PSMA-PET imaging at PSA values ≥ 0.5 ng/mL. 30.2% will perform PSMA-PET at ≥ 0.2 ng/mL. 16.3% and 2.3% will refer a patient to PSMA-PET at PSA levels ≥ 1.0 ng/mL and ≥ 2.0 ng/mL, respectively. 11.7% will perform PET regardless of the PSA value (see Fig. 2).

Median total dose for dose escalation of PET-positive local recurrence in EQD2(1.5 Gy) is 70 Gy (range: 56.89–85.71 Gy) with single doses of 2.00 Gy (range: 1.80–4.30 Gy). PET-positive pelvic lymph nodes are irradiated with a median total dose of 62.00 Gy (range: 52.61–80.00 Gy) in EQD2(1.5 Gy) with single doses of 2.00 Gy (range: 1.80–2.60 Gy) (see Table 2).

**Table 2:** Median total doses for dose escalation of PET-positive local recurrence and pelvic lymph nodes and median total dose for oligometastatic bone lesions

	Median total dose in EQD2(1.5 Gy) [Gy]	Median single dose [Gy]	RT technique
PET-positive local recurrence	70.00 (range: 56.89–85.71)	2.00 (range: 1.80–4.30)	SQB: 24.4% (21/86)
			SIB: 66.3% (57/86)
			No boost: 2.3% (2/86)
			Other: 7.0% (6/86), one with LDR-brachytherapy with 108 Gy
PET-positive pelvic LNs	62.00 (range: 52.61–80.00)	2.00 (range: 1.80–2.60)	SQB: 19.8% (13/84)
			SIB: 75.6% (65/84)
			No boost: 3.5% (3/86)
			Other: 1.2% (1/86) with a SBRT boost
Oligometastatic bone lesions	64.64 (range: 31.43–197.14)	5.00 (range: 1.80–20.00)	CRT: 51.4% (56/109)
			SBRT: 43.1% (47/109)
			No RT: 5.5% (6/109)

*RT* radiotherapy, *LN* lymph nodes, *EQD2(1.5 Gy)* equivalent dose at fractionation of 2 Gy with α/β = 1.5 Gy, *SQB* sequential boost, *SIB* simultaneous integrated boost, *LDR* low dose rate, *SBRT* stereotactic body radiotherapy, *CRT* conventional fractionated radiotherapy

**Fig. 3:**
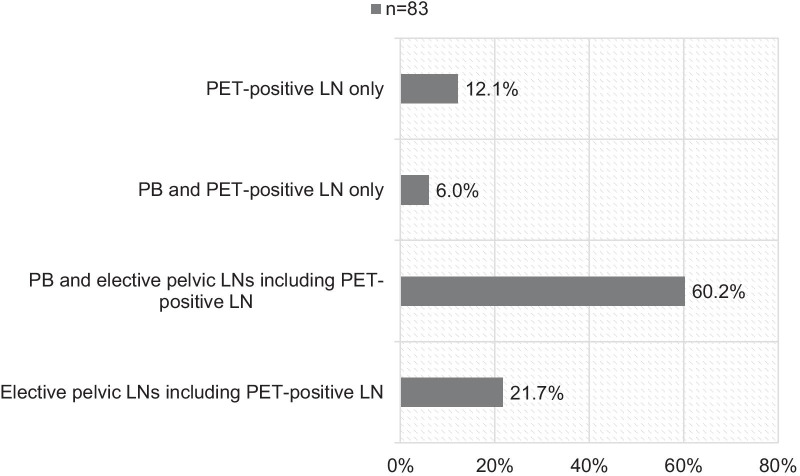
Treatment fields in cases of PET-positive LN only (n = 83, *PB* prostate bed, *LN* lymph node, *PET* positron emission tomography, *RT* radiotherapy, *PC* prostate cancer)

In cases of PET-positive pelvic lymph nodes only, most radiation oncologists will treat the elective pelvic lymphatic pathways including the PET-positive finding *and* the prostate bed. Figure 3 shows the treatment volumes in cases of PET-positive lymph nodes only without local recurrence.

Further, we asked whether the participants would prescribe additive ADT in cases of negative PET imaging before salvage RT. 61.6% (53/86) will recommend androgen deprivation therapy (ADT), while 26.8% (23/86) will not. 11.6% (10/86) will not recommend ADT regardless of the PET.

Moreover, 86.0% (74/86) of the radiation oncologists will initiate RT in case of a negative PET before salvage RT, while 14.0% (12/86) will postpone RT.

**Fig. 4:**
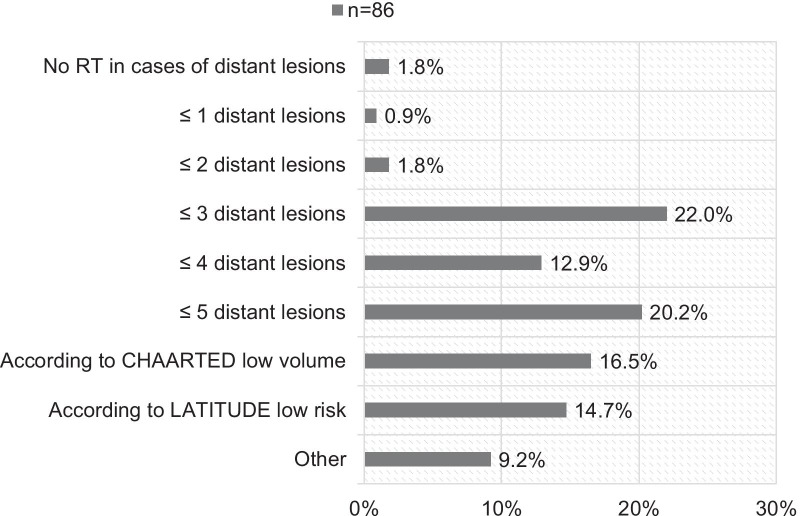
Participants’ definition of oligometastatic/oligorecurrent prostate cancer for local ablative RT. (Low volume according to CHAARTED [30], low risk according to LATITUDE [36], *RT* radiotherapy)

Most participants define ≤ 3 (22.0%, 24/109) or ≤ 5 (20.2%, 22/109) distant lesions as oligorecurrent/oligometastatic PC (see Fig. 4). 76.1% (83/109) of the interviewed radiation oncologists will recommend ADT in cases of RT for oligorecurrent/oligometastatic PC. Oligometastatic bone lesions are treated with conventionally fractionated RT in 51.4% (56/109) and with stereotactic body RT (SBRT) in 43.1% (47/109). 5.5% (6/109) of the interviewees do not treat bone lesions in the oligometastatic situation. Median total dose in EQD2(1.5 Gy) for bone metastases was 64.64 Gy (range: 31.43–197.14 Gy) with a median single dose of 5.00 Gy (range:1.80–20.00 Gy) (see Table 2).

## Discussion

We conducted a survey among German-speaking radiation oncologists to characterize the patterns of care for PET-based treatment for PC.

The data clearly show that PET imaging is widely used in centers of various institutional settings (78.9%). 96.5% of the radiation oncologists use PET for RT planning for PC; mostly for salvage RT (89.5%) or RT for oligometastatic/oligorecurrent PC (94.2%). Overall, most centers use PSMA-targeting agents (98.8%). Several series have shown that PSMA-ligand PET yields higher detection rate compared to choline-based PET-agents: A meta-analysis of Treglia et al. showed a clear benefit at PSA levels ≤ 1 ng/mL in patients with recurrent PC [8].

While PET/CT is the standard for image acquisition, hybrid PET/MR imaging is used by 34.9% of the participants in the present survey. Guberina et al. showed that PET/MRI and PET/CT have a similar detection rate, but PET/MRI is more accurate in cases of local recurrence [9]. However, average examination time for MRI and CT was 70 min versus 20 min favoring CT acquisition [9]. Due to longer scan duration, MRI is more cost-intensive compared to CT. A whole-body PET/CT with a pelvic MRI might be a good compromise to reduce examination time, while achieving a high detection rate.

A substantial amount of the participants (69.8%, 60/86) report about problems of cost coverage by health insurances. In Germany, in some areas PSMA-PET imaging is covered by health insurance after an individual decision process. Some centers have individual contracts with insurance companies, which made PSMA-PET imaging more accessible regionally. In February 2020 the GBA (Gemeinsamer Bundesausschuss) decided that PSMA-PET imaging should be covered by the health insurance companies in cases of PC recurrence after prior definitive treatment when offered as part of a so-called specialized out-patient care (ASV). Therefore, most of the participants use PET imaging in cases of rising PSA levels after definitive treatment in order to plan further treatment steps such as salvage RT or RT for oligorecurrent PC.

PSMA-PET at biochemical relapse is mostly performed at PSA levels ≥ 0.5 ng/mL (39.5%) or ≥ 0.2 ng/mL (30.2%). The European guideline [5] recommends PSMA-PET imaging at PSA levels > 0.2 ng/mL with a weak strength rating. Amongst others, Perera et al. showed that sensitivity of [68Ga]PSMA-PET increases with PSA levels. The authors reported rates of 45%, 59%, and 75% for PSA levels of 0.2–0.49, 0.5–0.99, and 1.0–1.99 ng/mL. For PSA < 0.2 ng/mL the scan positivity was only 33% [1]. The ideal PSA cut-off in cases of biochemical recurrence remains unclear; however, for patients with PSA levels over 0.2 ng/mL PSMA imaging should be discussed, if available. PSMA-PET can be considered as standard management for patients with rising PSA and should be performed as early as possible and when PSA levels allow. However, since some authors showed an improved outcome for very early salvage RT (< 0.2 ng/mL) [10–12] early treatment is desirable and should not be postponed.

Only about half of the radiation oncologists (46.5%) stated that they use PET imaging before definitive RT. Our own experience has shown that integration of [68Ga]PSMA-PET imaging into treatment planning for definitive RT in prostate cancer elevates the detection rate of lymph node spread; moreover, target volumes according to standard guidelines such as by RTOG would not cover affected lymph nodes adequately (up to 35.7% of all lymph nodes) without [68Ga]PSMA-PET imaging [13].

When PET imaging shows a local recurrence or pelvic lymph nodes most radiation oncologists use dose escalation protocols to target these PET-positive lesions. Simultaneous integrated boost (SIB) concepts are most widely used to escalate the radiation dose. The median total dose in EQD2(1.5 Gy) was 70.0 Gy (range: 56.89–85.71 Gy) for local recurrence and 62.00 Gy (range: 52.61–80.00 Gy) for lymph nodes. The National Comprehensive Cancer Network (NCCN) guideline recommends dose escalation in patients with clinically positive lymph nodes “as dose-volume histogram parameters allow” [14]. However, the dose prescription in lymph nodes is not clearly defined. The Australian and New Zealand guideline recommends a dose of over EQD2 60 Gy for positive lymph nodes in *definitive RT* [15]. A Singaporean guideline recommends 54–79.2 Gy, depending on the location of the irradiated lymph nodes for *definitive RT* [16]. Lately, Shakespeare et al. evaluated toxicity and outcome of PSMA-PET-based dose escalation for *definitive RT* with patients receiving 81 Gy in 45 fractions to the prostate and positive lymph nodes and 60 Gy in 45 fractions to the elective lymphatic pathways. This translates to an EQD2(1.5 Gy) of 76.37 Gy for positive lymph nodes. After 2 years, failure-free survival was 100% with acceptable reported acute and late toxicity [17].

In cases of *salvage RT*, a dose escalation for positive lymph nodes is also recommended by the Australian and New Zealand guideline; however, the optimal dose remains to be specified [18]. Schmidt-Hegemann et al. retrospectively evaluated PSMA-PET-based *salvage RT* and showed good results for a median total dose escalation of 60.8 Gy [19] and 61.6 Gy [20] to PET-positive lymph nodes while the elective pelvic lymph nodes were treated with a median dose of 50.4 Gy.

In summary, the optimal dose for macroscopic lymph nodes in cases of definitive and salvage RT remains a topic of discussion. However, the median EQD2(1.5 Gy) of 62.00 Gy in the present survey is in line with Australian/New Zealand guideline recommending doses of over 60 Gy. Bearing the dose–response relationship of PC in mind, higher doses of 70 Gy or more for lymph nodes (as described in the trial by Shakespear et al. [17]) might result in better outcome. Further investigation needs to be undertaken to evaluate the optimal dose.

The Australian and New Zealand guideline also recommends a dose escalation for macroscopic disease in the prostatic fossa with a dose of EQD2 70–74 Gy [18]. The rationale behind the dose escalation of macroscopic disease derives from the dose–response data for PC cells. The alpha/beta ratio for PC is described to be low [21]. Targets with a low alpha/beta ratio are more resistant to low doses compared to tissues with a high alpha/beta ratio. Therefore, higher total doses and hypofractionated schemes for the prostate have been increasingly used since improved radiation techniques allow for improved sparing of organs at risk.

In case of PET-positive lymph nodes without signs of local recurrence most participants (60.2%) will nevertheless treat the elective lymphatic pathways including the positive lymph node and the prostate bed. Only 21.7% will restrict the treatment field to the lymphatic pathways and the suspect lymph nodes. This remains a topic of discussion: Should we treat the prostate bed, if no macroscopic disease is present? And should we treat the elective lymph drainage if we can visualize lymph nodes affected by the disease? Panje et al. recently showed that among Swiss centers 58% of the participants additionally irradiate the prostate bed in cases of nodal oligorecurrence [22]. Zschaeck et al. showed in their survey that 92–100% will irradiate the prostate bed, pelvic lymphatic pathways and prescribe a boost to PSMA-positive lymph nodes [7]. To our knowledge, no data on RT of the pelvic lymph nodes ± prostate bed is available. But reducing the treatment field to the lymphatic pathways consequentially shrinks the dose to rectum and bladder and therefore might lower the risk of side effects. 12.1% of the participants in our survey will treat the PET-positive lymph nodes only, e.g. with SBRT. Panje et al. showed that fit patients with few lymph node metastases are considered to be the best candidates for SBRT [22]. Recently, Steuber et al. compared ADT to MDT such as SBRT or surgery for nodal only recurrence and showed that MDT improves cancer-specific survival [23]. Randomized data on elective lymph node RT versus SBRT of single lymph nodes is not available; however, SBRT could spare the patient from toxicity by shrinking the treatment field to the actual macroscopic disease. Salvage lymph node dissection in cases of nodal recurrence is an option. However, Schmidt-Hegemann et al. retrospectively compared PSMA-PET-based salvage RT versus salvage lymph node dissection for nodal recurrence and showed that RT was superior [24].

Most participants define oligometastatic or oligorecurrent PC as ≤ 3 or ≤ 5 distant lesions. There is no clear consensus of oligometastatic PC, and most authors use the definition of 3–5 lesions. The STOMP trial by Ost et al. [25] and the recent ORIOLE trial by Philipps et al. [26] used the cut-off of 3 lesions for comparing surveillance versus MDT and showed a better outcome for MDT. However, other series defined oligometastatic disease as ≤ 1 lesion [27], ≤ 4 lesions [28], or ≤ 5 lesions [29]. The definition of low volume metastatic disease by the CHAARTED trial is also popular among the participants. Low volume was defined as no visceral metastases and bone metastases confined to the vertebral column and pelvis [30]. However, the CHAARTED definition might not be suitable for the MDT approach as it allows an undefined number of bone metastases in the vertebra or pelvis. Additional ADT in cases of RT for oligometastatic PC is recommend by most of the participants (76.1%). ADT (± chemotherapy) is the standard management for metastatic PC. However, with the developments in RT (high precision SBRT) and imaging techniques (PSMA-PET imaging), MDT of the macroscopic tumor lesions is becoming another option for PC patients in the oligometastatic setting [25, 26]. This raises a crucial question: Can patients be spared from ADT and the side effects (at least for a certain time frame) with local approaches like MDT or is a combined concept with concurrent ADT to MDT the answer? In an earlier series, we showed that concurrent ADT to MDT improved biochemical failure-free survival. However, a large number of patients were spared from ADT initiation [31]. Siva et al. showed in the SABR trial that the ADT-free survival for patients who received PET-based MDT was 48% at 2 years [32]. Mazzola et al. compared patients with [68Ga]PSMA and [18F]Choline-based SBRT for oligorecurrent PC and showed that [68Ga]PSMA-based SBRT produced more ADT-free patients [33]. The STOMP trial [25] reported that the 5-year ADT-free survival was 8% for the observation group and 34% for the MDT group [34]. Consequentially, MDT might be an approach to spare patients from potential side effects of hormonal deprivation. However, patient selection is the key: The question of which patients might benefit from MDT alone and wich patients might profit from additional ADT should be clarified in further trials.

When bone metastases are treated in the oligometastatic setting, half of the participants use conventionally fractionated RT and the other half SBRT. This is surprising, since with SBRT high local ablative doses can be achieved. Ost et al. showed a high local control for patients treated with SBRT for oligometastatic PC with biological equivalent doses (BED) of > 100 Gy [35]. However, facilities without the technical ability for SBRT might opt for conventional fractionation.

Our study has certain limitations which are inherent to online questionnaires; we only asked general questions on RT doses in cases of PET-positive lesions. Participants were not able to differentiate doses dependent on location of the local recurrence or lymph nodes. Our goal was to present the individual opinions of radiation oncologists since it is inherent to online surveys that multiple answers from one institution cannot be prevented. Further, the data presented in this paper reflect the day-to-day situation in German-speaking countries but might be applicable to other regions as well. The data is a valuable addendum to all other published information on use and recommendations of PET imaging for PC and focusses on the end of the treatment loop—the way a new treatment and imaging modality is accepted and integrated into daily clinical practice.

## Conclusion

PSMA-PET imaging has emerged to be a key pillar in RT for PC and has been included into daily clinical practice. In this analysis focusing on the use among German-speaking radiation oncologists, procedures and treatment patterns differ, reflecting an often-observed phenomenon of a highly specialized technique in day-to-day use. Therefore, further trials and more specific guidelines for PET-based RT are essentially required, as well as education on the use and limitations of molecular imaging for RT planning. In that regard, multidisciplinary research and working groups are essential to foster interaction and to promote standardization.

## Supplementary Information

**Additional file 1.** Survey_PET.pdf. Questions of the survey concerning PET-based radiotherapy.

## Data Availability

The datasets generated during and/or analyzed during the current study are available from the corresponding author on reasonable request.

## Abbreviations

(3D-)CRT: Three-dimensional conventional radiotherapy
ADT: Androgen deprivation therapy
ASV: Specialized out-patient care
BED: Biological equivalent doses
CT: Computed tomography
DEGRO: German Society of Radiation Oncology
EQD2(1.5 Gy): Equivalent dose at fractionation of 2 Gy with α/β prostate = 1.5 Gy
Ga: Gallium
GBA: Gemeinsamer Bundesausschuss
Gy: Gray
IGRT: Image-guided radiotherapy
IMRT: Intensity-modulated radiotherapy
LDR: Low dose rate
LN: Lymph nodes
MDT: Metastasis-directed therapy
MRI: Magnetic resonance imaging
N: Number
NCCN: National Comprehensive Cancer Network
PC: Prostate cancer
PET: Positron emission tomography
PSA: Prostate-specific antigen
PSMA: Prostate-specific membrane antigen
RT: Radiotherapy
SBRT: Stereotactic body radiotherapy
SIB: Simultaneous integrated boost
VMAT: Volumetric arc therapy

## References

[1] Perera M, Papa N, Christidis D, Wetherell D, Hofman MS, Murphy DG (2016). Sensitivity, specificity, and predictors of positive (68)Ga-prostate-specific membrane antigen positron emission tomography in advanced prostate cancer: a systematic review and meta-analysis. Eur Urol.

[2] Frenzel T, Tienken M, Abel M, Berliner C, Klutmann S, Beyersdorff D (2018). The impact of [68Ga]PSMA I&T PET/CT on radiotherapy planning in patients with prostate cancer. Strahlenther Onkol.

[3] Hurmuz P, Onal C, Ozyigit G, Igdem S, Atalar B, Sayan H (2020). Treatment outcomes of metastasis-directed treatment using 68Ga-PSMA-PET/CT for oligometastatic or oligorecurrent prostate cancer: Turkish Society for Radiation Oncology group study (TROD 09–002). Strahlenther Onkol.

[4] Hofman MS, Lawrentschuk N, Francis RJ, Tang C, Vela I, Thomas P (2020). Prostate-specific membrane antigen PET-CT in patients with high-risk prostate cancer before curative-intent surgery or radiotherapy (proPSMA): a prospective, randomised, multi-centre study. Lancet (London, England).

[5] Mottet N, Bellmunt J, Bolla M, Briers E, Cumberbatch MG, De Santis M (2017). EAU-ESTRO-SIOG guidelines on prostate cancer. Part 1: screening, diagnosis, and local treatment with curative intent. Eur Urol.

[6] Leitlinienprogramm Onkologie (Deutsche Krebsgesellschaft DK, AWMF). Interdisziplinäre Leitlinie der Qualität S3 zur Früherkennung, Diagnose und Therapie der verschiedenen Stadien des Prostatakarzinoms, Langversion 5.1, AWMF Registernummer: 043/022OL. 2019. http://www.leitlinienprogramm-onkologie.de/leitlinien/prostatakarzinom/. Accessed 05 Apr 2020.

[7] Zschaeck S, Lohaus F, Beck M, Habl G, Kroeze S, Zamboglou C (2018). PSMA-PET based radiotherapy: a review of initial experiences, survey on current practice and future perspectives. Radiat Oncol (London, England).

[8] Treglia G, Pereira Mestre R, Ferrari M, Bosetti DG, Pascale M, Oikonomou E (2019). Radiolabelled choline versus PSMA PET/CT in prostate cancer restaging: a meta-analysis. Am J Nucl Med Mol Imaging.

[9] Guberina N, Hetkamp P, Ruebben H, Fendler W, Grueneisen J, Suntharalingam S (2019). Whole-body integrated [(68)Ga]PSMA-11-PET/MR imaging in patients with recurrent prostate cancer: comparison with whole-body PET/CT as the standard of reference. Mol Imaging Biol.

[10] Bartkowiak D, Bottke D, Thamm R, Siegmann A, Hinkelbein W, Wiegel T (2016). The PSA-response to salvage radiotherapy after radical prostatectomy correlates with freedom from progression and overall survival. Radiother Oncol.

[11] Abugharib A, Jackson WC, Tumati V, Dess RT, Lee JY, Zhao SG (2017). Very early salvage radiotherapy improves distant metastasis-free survival. J Urol.

[12] Tendulkar RD, Agrawal S, Gao T, Efstathiou JA, Pisansky TM, Michalski JM (2016). Contemporary update of a multi-institutional predictive nomogram for salvage radiotherapy after radical prostatectomy. J Clin Oncol.

[13] Schiller K, Devecka M, Maurer T, Eiber M, Gschwend J, Schwaiger M (2018). Impact of (68)Ga-PSMA-PET imaging on target volume definition and guidelines in radiation oncology—a patterns of failure analysis in patients with primary diagnosis of prostate cancer. Radiat Oncol.

[14] Mohler JL, Antonarakis ES, Armstrong AJ, D'Amico AV, Davis BJ, Dorff T (2019). Prostate cancer, Version 2.2019, NCCN clinical practice guidelines in oncology. J Natl Compr Cancer Netw JNCCN.

[15] Lieng H, Kneebone A, Hayden AJ, Christie DRH, Davis BJ, Eade TN (2019). Radiotherapy for node-positive prostate cancer: 2019 recommendations of the Australian and New Zealand Radiation Oncology Genito-Urinary group. Radiother Oncol.

[16] Sim HG, Lim KH, Tay MH, Chong KT, Chiong E (2013). Guidelines on management of prostate cancer. Ann Acad Med Singap.

[17] Shakespeare TP, Eggert E, Wood M, Westhuyzen J, Turnbull K, Rutherford N (2019). PSMA-PET guided dose-escalated volumetric arc therapy (VMAT) for newly diagnosed lymph node positive prostate cancer: efficacy and toxicity outcomes at two years. Radiother Oncol.

[18] Lieng H, Hayden AJ, Christie DRH, Davis BJ, Eade TN, Emmett L (2018). Radiotherapy for recurrent prostate cancer: 2018 recommendations of the Australian and New Zealand Radiation Oncology Genito-Urinary group. Radiother Oncol.

[19] Schmidt-Hegemann N-S, Stief C, Kim T-H, Eze C, Kirste S, Strouthos I (2018). Outcome after PSMA PET/CT based salvage radiotherapy in patients with biochemical recurrence after radical prostatectomy: a bi-institutional retrospective analysis. J Nucl Med.

[20] Schmidt-Hegemann NS, Fendler WP, Ilhan H, Herlemann A, Buchner A, Stief C (2018). Outcome after PSMA PET/CT based radiotherapy in patients with biochemical persistence or recurrence after radical prostatectomy. Radiat Oncol.

[21] Vogelius IR, Bentzen SM (2013). Meta-analysis of the alpha/beta ratio for prostate cancer in the presence of an overall time factor: bad news, good news, or no news?. Int J Radiat Oncol Biol Phys.

[22] Panje C, Zilli T, Pra AD, Arnold W, Brouwer K, Garcia Schüler HI (2019). Radiotherapy for pelvic nodal recurrences after radical prostatectomy: patient selection in clinical practice. Radiat Oncol.

[23] Steuber T, Jilg C, Tennstedt P, De Bruycker A, Tilki D, Decaestecker K (2019). Standard of care versus metastases-directed therapy for PET-detected nodal oligorecurrent prostate cancer following multimodality treatment: a multi-institutional case-control study. Eur Urol Focus.

[24] Schmidt-Hegemann N-S, Buchner A, Eze C, Rogowski P, Schaefer C, Ilhan H (2020). PSMA-positive nodal recurrence in prostate cancer. Strahlenther Onkol.

[25] Ost P, Reynders D, Decaestecker K, Fonteyne V, Lumen N, De Bruycker A (2018). Surveillance or metastasis-directed therapy for oligometastatic prostate cancer recurrence: a prospective, randomized, multicenter phase II trial. J Clin Oncol.

[26] Phillips R, Shi WY, Deek M, Radwan N, Lim SJ, Antonarakis ES (2020). Outcomes of observation vs stereotactic ablative radiation for oligometastatic prostate cancer: the ORIOLE phase 2 randomized clinical trial. JAMA Oncol.

[27] Jereczek-Fossa BA, Piperno G, Ronchi S, Catalano G, Fodor C, Cambria R (2014). Linac-based stereotactic body radiotherapy for oligometastatic patients with single abdominal lymph node recurrent cancer. Am J Clin Oncol.

[28] Schick U, Jorcano S, Nouet P, Rouzaud M, Vees H, Zilli T (2013). Androgen deprivation and high-dose radiotherapy for oligometastatic prostate cancer patients with less than five regional and/or distant metastases. Acta Oncol.

[29] Singh D, Yi WS, Brasacchio RA, Muhs AG, Smudzin T, Williams JP (2004). Is there a favorable subset of patients with prostate cancer who develop oligometastases?. Int J Radiat Oncol Biol Phys.

[30] Sweeney CJ, Chen YH, Carducci M, Liu G, Jarrard DF, Eisenberger M (2015). Chemohormonal therapy in metastatic hormone-sensitive prostate cancer. N Engl J Med.

[31] Kroeze SGC, Henkenberens C, Schmidt-Hegemann NS, Vogel MME, Kirste S, Becker J (2019). Prostate-specific membrane antigen positron emission tomography-detected oligorecurrent prostate cancer treated with metastases-directed radiotherapy: role of addition and duration of androgen deprivation. Eur Urol Focus.

[32] Siva S, Bressel M, Murphy DG, Shaw M, Chander S, Violet J (2018). Stereotactic abative body radiotherapy (SABR) for oligometastatic prostate cancer: a prospective clinical trial. Eur Urol.

[33] Mazzola R, Francolini G, Triggiani L, Napoli G, Cuccia F, Nicosia L (2020). Metastasis-directed therapy (SBRT) guided by PET-CT (18)F-CHOLINE versus PET-CT (68)Ga-PSMA in castration-sensitive oligorecurrent prostate cancer: a comparative analysis of effectiveness. Clin Genitourin Cancer.

[34] Ost P, Reynders D, Decaestecker K, Fonteyne V, Lumen N, De Bruycker A (2020). Surveillance or metastasis-directed therapy for oligometastatic prostate cancer recurrence (STOMP): five-year results of a randomized phase II trial. J Clin Oncol.

[35] Ost P, Jereczek-Fossa BA, As NV, Zilli T, Muacevic A, Olivier K (2016). Progression-free survival following stereotactic body radiotherapy for oligometastatic prostate cancer treatment-naive recurrence: a multi-institutional analysis. Eur Urol.

[36] Fizazi K, Tran N, Fein L, Matsubara N, Rodriguez-Antolin A, Alekseev BY (2017). Abiraterone plus prednisone in metastatic, castration-sensitive prostate cancer. N Engl J Med.

