# Supplementary material for: A survey among German-speaking radiation oncologists on PET-based radiotherapy of prostate cancer
Source: Radiat Oncol. 2021 May 1;16:82. doi: 10.1186/s13014-021-01811-8 (PMC8088662; doi:10.1186/s13014-021-01811-8)
Supplement: Supplementary file 1 — Additional file 1. Survey_PET.pdf. Questions of the survey concerning PET-based radiotherapy. [file 13014_2021_1811_MOESM1_ESM.pdf]

1. In welcher Einrichtung sind Sie tätig?
    a. Einzel- oder Gemeinschaftspraxis
    b. Medizinisches Versorgungszentrum (MVZ)
    c. Nicht-universitäre Klinik
    d. Universitätsklinik

2. In welcher Position sind Sie tätig?
    a. Arzt*Ärztin in Weiterbildung
    b. Angestellter*e Facharzt*ärztin
    c. Inhaber*in
    d. Medizinisch-technische*r Radiologieassistent*in
    e. Medizinphysik-Experte*in
    f. Anderes:

3. In welcher Position sind Sie tätig?
    a. Assistenzarzt*ärztin
    b. Facharzt*ärztin
    c. Oberarzt*ärztin
    d. Chefarzt*ärztin
    e. Medizinisch-technische*r Radiologieassistent*in
    f. Medizinphysik-Experte*in
    g. Anderes:

4. Welche Techniken stehen Ihnen zur Verfügung? (Mehrfachauswahl möglich)
    a. 3D-RT
    b. IMRT/VMAT
    c. Helikale IMRT
    d. IGRT
    e. Stereotaxie
    f. Protonen/Schwerionen
    g. Brachytherapie
    h. Andere:

Die folgenden Fragen beziehen sich auf die Planung der Radiotherapie eines Prostatakarzinoms mit Hilfe der PET-Bildgebung.

17. Steht Ihnen in Ihrer Einrichtung eine PET-Bildgebung zur Verfügung?
    a. Ja, eine PET-Bildgebung steht direkt in der Klinik/Einrichtung zur Verfügung
    b. Ja, eine PET-Bildgebung steht über einen externe Kooperationspartner zur Verfügung
    c. Nein

18. Verwenden Sie die PET-Bildgebung zur Therapieplanung?
    a. Ja
    b. Nein

19. Welche Tracer werden für die PET-Bildgebung bei Patienten mit Prostatakarzinom verwendet? (Mehrfachauswahl möglich)
    a. Cholin Tracer

    b. PSMA Tracer
    c. Sonstige:

20. Welche Bildmodalitäten für die PET-Bildgebung werden verwendet? (Mehrfachauswahl möglich)
    a. CT
    b. MRT

21. Bestehen Probleme bei der Übernahme der Kosten durch die Krankenkasse für eine PET-Bildgebung?
    a. Ja
    b. Nein

22. In welchen Situationen verwenden Sie die PET-Bildgebung zur Therapieplanung? (Mehfachauswahl)
    a. Definitive Radiotherapie des Prostatakarzinom
    b. Adjuvante Radiotherapie des Prostatakarzinom
    c. Salvage-Radiotherapie des Prostatakarzinoms
    d. Radiotherapie des olgiometastasierten Prostatakarzinom
    e. Andere:

23. Wird im Falle eines PET-positiven Lokalrezidivs, dieses mit einem Boost bei einer perkutanen Salvage-Radiotherapie behandelt?
    a. Ja, mit einem simultan-integrierten Boost (SIB)
    b. Ja, mit einem sequentiellen Boost
    c. Nein
    d. Andere

24. Mit welcher Gesamtdosis wird das Lokalrezidiv im Boost in Ihrer Einrichtung überwiegend behandelt?
    a. Gesamtdosis
    b. Einzeldosis

25. Wird im Falle von PET-positiven pelvine Lymphknoten, diese mit einem Boost bei einer definitiven oder postoperativen Radiotherapie behandelt?
    a. Ja, mit einem simultan-integrierten Boost (SIB)
    b. Ja, mit einem sequentiellen Boost
    c. Nein
    d. Andere

26. Mit welcher Gesamtdosis werden PET-positive pelvine Lymphknoten im Boost in Ihrer Einrichtung überwiegend behandelt?
    a. Gesamtdosis
    b. Einzeldosis

27. Wie erfolgt die Behandlung, wenn sich vor einer Salvage-Radiotherapie in der Bildgebung nur pelvine Lymphknotenmetastasen ohne eine Lokalrezidiv in der Prostataloge zeigen?
    a. Radiotherapie der einzelnen pelvinen Lymphknotenmetastase
    b. Radiotherapie des elektiven pelvinen Lymphabflussweges inklusive der einzelnen pelvinen Lymphknotenmetastasen
    c. Radiotherapie der Prostataloge und der einzelnen pelvinen Lymphabflusswege
    d. Radiotherapie der Prostataloge und des elektiven pelvinen Lymphabflussweges inklusive der einzelnen pelvinen Lymphknotenmetastasen

28. Hat eine negative PET-Bildgebung Auswirkung auf die Indikation zur zusätzlichen Hormontherapie bei der Salvage-Radiotherapie?
    a. Ja, bei negativem PET würde ich auf eine zusätzliche Hormontherapie verzichten.
    b. Nein, bei negativem PET würde ich trotzdem eine zusätzliche Hormontherapie in Erwägung ziehen.
    c. Ich empfehle keine Hormontherapie in der Salvage-Situation.

29. Würden Sie bei einer negativen PET-Bildgebung aber steigenden PSA-Wert eine perkutane Salvage-Radiotherapie durchführen?
    a. Ja, ein negative PET-Bildgebung sollte eine Salvage-Radiotherapie nicht verzögern.
    b. Ein Abwarten des nächsten PSA-Werts und ein neue PET-Bildgebung sind gerechtfertigt.

30. Bei welchem PSA-Wert würden Sie eine PET-Bildgebung bei der perkutanen Salvage-Radiotherapie zur Therapieplanung durchführen lassen?
    a. Unabhängig vom PSA-Wert
    b. Ab 0,2 ng/ml
    c. Ab 0,5 ng/ml
    d. Ab 1,0 ng/ml
    e. Ab 2,0 ng/ml

Die folgenden Fragen beziehen sich auf die Therapie des oligometastasierten/oligorezidivierten Prostatakarzinom

31. Welche Anzahl von Fernmetastasen würden Sie als oligometastasiertes/oligorezidiviertes Prostatakarzinom bezeichnen und deswegen die Fernmetastasen lokal ablativ in individueller kurativer Intention behandeln?
    a. Bei Fernmetastasierung erfolgt in meiner Einrichtung keine lokal ablative Behandlung in individueller kurativer Intention
    b. ≤1
    c. ≤2
    d. ≤3
    e. ≤4
    f. ≤5
    g. Low Volume nach CHAARTED[1]
    h. Low Risk nach LATITUDE[2]
    i. Andere:

[1]<4 Knochenmetastasen beschränkt auf Wirbelkörper/Becken und keine viszeralen Metastasen
[2]mindestens 2 Kriterien: <3 Knochenmetastasen, Gleason Score <8, keine viszeralen Metastasen

32. Sollten Patienten in der oligometastasierten/oligorezidivierten Situation eine zusätzliche Hormontherapie zur Radiotherapie erhalten?
    a. Ja
    b. Nein

33. Wie behandeln Sie überwiegend Knochenmetastasen in der oligometastasierten/oligorezidivierten Situation lokal ablativ?

a. Normofraktionierte Konzepte
b. Körperstereotaxie (SBRT)
c. Knochenmetastasen werden in der oligometastasierten/oligorezidivierten Situation in meiner Einrichtung nicht lokal ablativ behandelt.

34. Welche Dosis wird bei einer SBRT einer Knochenmetastase verschrieben?
    a. Gesamtdosis
    b. Einzeldosis

35. Wird in Ihrer Einrichtung eine Re-Bestrahlung des Beckens bei Lokalrezidiv oder positiven Lymphknoten angeboten? (Mehrfachauswahl möglich)
    a. Ja, bei Zustand nach postoperativer Radiotherapie
    b. Ja, bei Zustand nach definitiver Radiotherapie
    c. Nein
